# Immunohistochemical Analysis of IL-6, IL-8/CXCR2 Axis,  ^Tyr^p-STAT-3, and SOCS-3 in Lymph Nodes from Patients with Chronic Lymphocytic Leukemia: Correlation between Microvascular Characteristics and Prognostic Significance

**DOI:** 10.1155/2014/251479

**Published:** 2014-05-05

**Authors:** Georgia Levidou, Sotirios Sachanas, Gerassimos A. Pangalis, Christina Kalpadakis, Xanthi Yiakoumis, Maria Moschogiannis, Athanasia Sepsa, Eleftheria Lakiotaki, Vassilis Milionis, Marie-Christine Kyrtsonis, Theodoros P. Vassilakopoulos, Pantelis Tsirkinidis, Flora Kontopidou, Styliani Kokoris, Marina Siakantaris, Maria Angelopoulou, Helen Papadaki, Nikolaos Kavantzas, Panayiotis Panayiotidis, Efstratios Patsouris, Penelope Korkolopoulou

**Affiliations:** ^1^First Department of Pathology, Laikon Hospital, University of Athens, Medical School, 75 Mikras Asias street, 11527 Athens, Greece; ^2^Athens Medical Center-Psychicon Branch, 15451 Athens, Greece; ^3^Hematology Department, University of Crete, 70013 Heraklion, Greece; ^4^Hematology Department, University of Athens, Laikon General Hospital, 11527 Athens, Greece; ^5^Hematology Department, University of Athens, Ipokration General Hospital, 11527 Athens, Greece; ^6^Hematology Department, University of Athens, Attikon General Hospital, 12461 Athens, Greece; ^7^First Department of Internal Medicine, University of Athens, Laikon General Hospital, 11527 Athens, Greece

## Abstract

A number of studies have looked into the pathophysiological role of angiogenesis in CLL, but the results have often been inconsistent. We aimed to gain direct insight into the angiogenic process in lymph nodes involved by CLL, focusing on proangiogenic cytokines and microvessel morphometry. The tissue levels of VEGF, Th-2 cytokines IL-6 and IL-8, IL-8 receptor CXCR2, and tyrosine p-STAT-3/SOCS-3 axis modulating cytokine expression were evaluated immunohistochemically in 62 CLL/SLL cases. Microvascular characteristics were evaluated by image analysis. Results were analyzed with regard to clinicopathological characteristics. Proliferation centers (PCs) were less well vascularised compared to non-PC areas. IL-8 and CXCR2 expression was distinctly uncommon as opposed to IL-6, VEGF and SOCS-3, which were detected in the vast majority of cases. The latter two molecule expressions were more pronounced in the PCs in *∼*40% of the cases. p-STAT-3 immunoreactivity was recorded in 66.67% of the cases with a predilection for PCs. Microvessel morphometry was unrelated to proangiogenic cytokines, p-STAT-3, SOCS-3, or survival. Microvascular caliber and VEGF expression were higher in Binet stage A, whereasIL-6 expression was higher in stage C. VEGF and p-STAT-3 exerted a favorable effect on progression, which remained significant in multivariate analysis, thereby constituting potential outcome predictors in CLL patients.

## 1. Introduction

Accumulating evidence suggests that angiogenesis is of vital importance for the development and progression of several malignancies, including chronic lymphocytic leukemia (CLL) [1, 2]. A variety of abnormalities in the angiogenic profile of CLL patients have been reported including elevated serum levels of certain proangiogenic cytokines and vascular endothelial growth factor (VEGF) [3, 4], whereas current belief holds that tumor cells themselves are an important source of proangiogenic factors [5]. Angiogenic activation, however, is a complex biological process regulated by a delicate balance and interaction between soluble or membrane bound pro- and antiangiogenic factors.

The Th-2 cytokine interleukin 6 (IL-6), one of the most ubiquitously deregulated cytokines in human malignancies, harbors a basic role in the immune response, inflammation, and hemopoiesis and is a potent promoter of angiogenesis modulating VEGF expression [6, 7]. IL-6 signals through a cell-surface cytokine receptor, which activates the tyrosine kinase JAK and ultimately the signal transducer and activator of transcription (STAT)-3 [8]. STAT-3 becomes activated (i.e., phosphorylated at tyrosine 705) in response to cytokines, growth factors, and extracellular signals [9]. However, STAT-3 is constitutively activated in CLL cells through phosphorylation at serine 727 [10]. Phosphorylated (p-) STAT-3 induces tumor angiogenesis by upregulating the expression of VEGF and modulates immune functions facilitating immune evasion. Indeed, it is not surprising that several studies point towards STAT-3 as a promising target for anticancer therapy [11–14]. Importantly, uncontrolled cytokine-driven STAT activation could have disastrous biologic consequences. Accordingly, the suppressors of the cytokine signaling (SOCS) proteins, namely, SOCS1–SOCS7 and the SH2 domain-containing proteins, which are the only known inhibitors of STAT pathway, have drawn considerable attention [15]. STAT-3, in particular, induces SOCS-3 which acts as a negative regulator of the aforementioned pathway and opposes its proliferative and antiapoptotic effect [16, 17].

Proinflammatory chemokine IL-8, another Th-2 cytokine, displays equipotent angiogenic properties with IL-6 via its Glu-Leu-Arg (ELR) motif [18, 19]. The biologic effects of IL-8 are mediated by two highly related G protein-coupled receptors, namely, chemokine (C-X-C motif) receptor 1 (CXCR1) and CXCR2. Despite many functional similarities between these two receptors, CXCR2 is the prime receptor mediating ELR+ chemokines' angiogenic effects [20, 21].

In the present study, we aimed for the first time to evaluate immunohistochemically the tissue levels of proangiogenic factors, that is, VEGF, IL-6, IL-8 along with IL-8 receptor CXCR2, tyrosine p-STAT-3, and SOCS-3 in the lymph nodes, in a series of patients with CLL/SLL. We were particularly interested in any differences which might exist in the expression within and outside the proliferating centers (PCs), as well as in any correlation with patients' clinicopathological and biological characteristics. Additionally, we examined the relationships of these molecules with microvascular characteristics of lymph nodes aiming to gain insight into their potential implication in the angiogenic process. Finally, we examined the potential prognostic effect of these molecules and microvascular characteristics in patients' overall survival (OS), time to first treatment (TFT), and time to progression (TTP).

## 2. Materials and Methods

### 2.1. Patients

This is a study of 62 patients fulfilling the diagnostic criteria of CLL/SLL according to the 2008 World Health Classification of Tumors of Hematopoietic and Lymphoid Tissue [22] for whom archival primary lymph node tissue material at diagnosis or at least prior to treatment initiation was available.

The characteristics of the patients enrolled in the present study are presented in Table 1. Mutational IgVH status was analyzed in 16 patients, while FISH analysis for 11q and 17p deletion was performed in 18 and 22 patients, respectively, for whom peripheral blood samples at diagnosis were available. IgVH clonality analysis was done in DNA extracts from patients' blood samples using the suggested primers by the European Collaborative Study BIOMED 2 [23]. FISH studies were performed in peripheral blood lymphocytes with the LSI p53/LSI ATM multicolor probe sets provided by Vysis (Downers Grove, IL, USA) using defined cutoff levels [24]. CD38 expression was analyzed by flow cytometry in peripheral blood and was considered to increase when ≥30% positive cells were encountered, conforming to published convention [25].

### 2.2. Immunohistochemical Analysis

Serial sections were used in order to be able to make comparisons among the immunostained slides as well as with the respective HE sections and ensure that the PC areas were correctly identified. Immunostaining for IL-8, IL-6, CXCR2, SOCS-3, tyrosine p-STAT-3, and VEGF was performed on paraffin-embedded 4 *μ*m sections using the two-step peroxidase conjugated polymer technique (DAKO Envision kit, DAKO, Carpinteria, CA). The primary antibodies used are listed in Table 2. In addition, information regarding the immunohistochemical expression of Ki67 index, Zap 70, Fas, FasL, c-FLIP within and outside the PCs was available in 44 lymph node cases from our previous investigation [26].

Evaluation of immunostained slides stained with IL-8, IL-6, CXCR2, SOCS-3, tyrosine p-STAT-3, and VEGF was performed blindly using light microscopy. Evaluation of staining intensity was based on the comparison of the intensity observed in the neoplastic cells when compared with the one observed in endothelial or plasma cells and was categorized into three categories as follows: faint (1-less intense staining than the one observed in endothelial/plasma cells), moderate (2-intensity equal to the one observed in endothelial/plasma cells), and intense staining (3-more intense staining than the one observed in endothelial/plasma cells). A histoscore (H-score) representing the percentage of stained lymphoid cells multiplied by staining intensity was calculated for each molecule.

The microvascular characteristics were evaluated on CD34 immunostained slides. Microvessel density (MVD), total vascular area (TVA), and several other size- and shape-related parameters were quantified in the region of most intense vascularization as well as within and outside the PCs, using the computerized image analysis software Image Pro software v5.1 (Media Cybernetics Inc.) on a Pentium III PC, as described previously [27]. For each countable microvessel, several morphometric parameters were automatically established: major axis length (i.e., the distance between the two points along the vessel periphery that are further apart), minor axis length (i.e., the longest axis perpendicular to major axis formed by two points along the vessel periphery), perimeter, area (luminal plus endothelial cell area), Feret diameter $\left(\sqrt{4*\text{area}/\pi }\right)$, shape factor (4*π*∗area/perimeter^2^), compactness (perimeter^2^/area), microvessel density(MVD)(i.e., the total count of microvessels per optical field), and total vascular area (TVA) (i.e., the total area occupied by microvessels). For each case, the mean value of major and minor axis length, area, perimeter, Feret diameter, shape factor and compactness along with MVD and TVA were recorded for statistical analysis.

### 2.3. Statistical Analysis

In the basic statistical analysis IL-6, SOCS-3, tyrosine p-STAT-3, and VEGF expression and microvascular characteristics were treated as continuous variables. On the other hand, IL-8 and CXCR2 were treated as categorical variables according to the presence or absence of immunoreactivity. Associations with clinicopathological parameters and microvascular characteristics were tested using nonparametric tests (Spearman correlation coefficient, Mann-Whitney* U* test, and Kruskal-Wallis ANOVA), as appropriate.

Survival analysis for OS, TFT, and TTP was performed using death of disease, first therapeutic approach, and disease progression as an end-point, respectively. The effect of various clinicopathological parameters on clinical outcome (OS, TFT and TTP) was assessed by plotting survival curves according to the Kaplan-Meier method and comparing groups using the log-rank test. Numerical variables were categorized on the basis of the median values. Multivariate analysis was performed using stepwise Cox's proportional hazard estimation model with forward and backward selection of variables. Due to the small number of events encountered in our cohort, in order to ensure the power of our analysis, only those parameters for which there were not any significant missing data and presented with a statistically significant result in univariate survival analysis were introduced in the multivariate models. Statistical calculations were performed using the Statistical package STATA 11.0 for Windows (Stata Corp. College Station, TX, USA). All results with a two-sided* P* level ≤0.05 were considered statistically significant.

## 3. Results

### 3.1. Microvascular Characteristics within and outside the PCs

**Figure 1:**
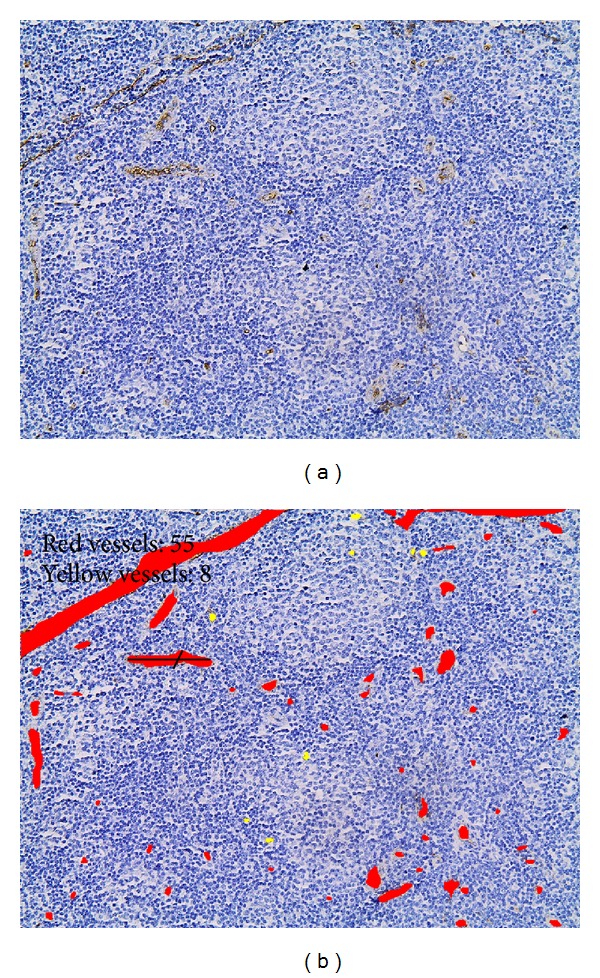
(a) Immunohistochemical staining of CD34 in a CLL case. (b) Same field as in (a). The outline of each vessel is traced; the red layer represents the section area of each vessel outside the PCs, whereas the yellow layer represents the section area of each vessel within the PCs. PCs display clearly higher MVD and TVA and rounder vessels when compared to non-PC areas. (PC: proliferation centers).

**Figure 2:**
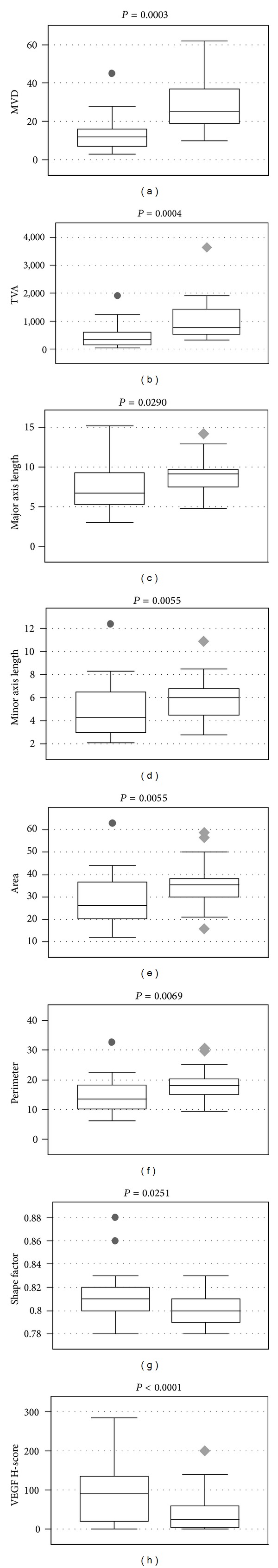
Box plots illustrating the lower levels of MVD, major axis length, minor axis length, area, perimeter, and TVA and the higher levels of shape factor and VEGF H-score in the PCs when compared to the non-PC areas.

Microvascular characteristics within and outside the PCs are shown in Table 3 and Figure 1. MVD, major axis length, minor axis length, area, perimeter, and TVA were lower in the PCs when compared to the non-PC areas (Wilcoxon matched pairs signed-rank test, *P* = 0.0003 for MVD, *P* = 0.0073 for major axis length, *P* = 0.0290 for minor axis length, *P* = 0.0055 for area, *P* = 0.0069 for perimeter, and *P* = 0.0004 for TVA, Figures 2(a)–2(f)). On the contrary shape factor was higher within the PC areas when compared with the non-PC areas (Wilcoxon matched pairs signed-rank test, *P* = 0.0251, Figure 2(g)), as illustrated by the preponderance of rounder vessels sections in the former and more flattened ones in the latter (Figure 1).

### 3.2. Immunohistochemical Expression of IL-6, IL-8, CXCR2, SOCS-3, Tyrosine p-STAT-3, and VEGF in CLL

**Figure 3:**
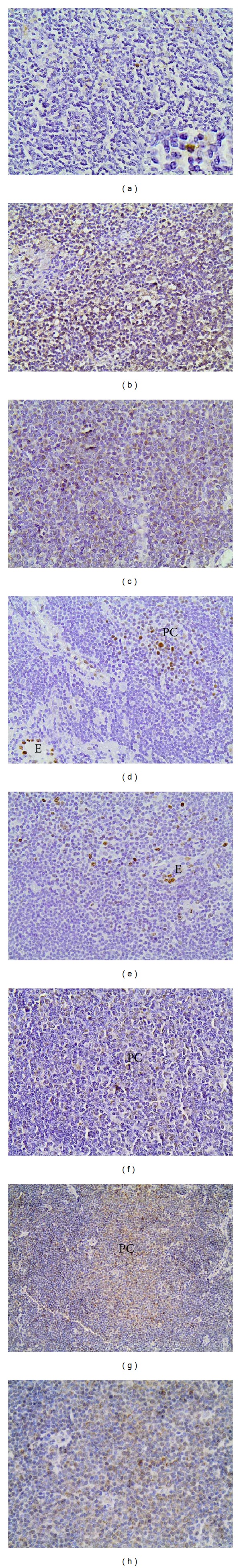
Immunohistochemical expression of IL-8 (a), CXCR2 (b), IL-6 (c), tyrosine p-STAT-3 (d, e), SOCS-3 (f), and VEGF (g, h) in lymph nodes from CLL patients. (a) A CLL case with very few scattered IL-8 positive lymphoid cells. The inset shows an IL-8 positive cell in a higher magnification. Note the positive endothelial cells. (b) CXCR2 expression in a CLL lymph node. (c) IL-6 expression homogeneously distributed throughout the lymphoid tissue. (d) Tyrosine p-STAT-3 in a case showing a more pronounced immunoreactivity in the PCs. Note that endothelial cells are strongly positive. (e) Scattered tyrosine p-STAT-3 positive lymphoid cells along with positive endothelial cells in a CLL case. (f) Higher SOCS-3 expression in the PC in a CLL case. (g) (h) Pronounced VEGF immunoexpression in the PCs compared with the area outside the PCs. Higher magnification (h) showing the cytoplasmic immunoreactivity of VEGF. (PC: proliferation centers; E: endothelial cells).

IL-8 and CXCR2 expression was detected only in 5/51 (9.80%) and 7/51 (13.21%) of the examined cases, respectively (Table 4, Figures 3(a) and 3(b)). On the contrary, IL-6 expression was detected in 49/51 (96.08%) of the examined cases (Table 4, Figure 3(c)). Immunoreactivity for all antibodies was cytoplasmic and did not show a predilection for PCs. IL-6 immunostaining was consistently observed in lymphocytes, prolymphocytes, and paraimmunoblasts. Plasma cells were also positive for IL-6 and served as internal positive controls in negative cases.

Tyrosine p-STAT-3 expression was nuclear and was detected in 36/51 (66.67%) of the examined cases (Table 4, Figures 3(d) and 3(e)). In 21/35 (66.67%) positive cases, immunoreactivity was mainly observed in the PCs (Figure 3(d)). In the remaining 14 cases either there was a paucity of PCs (3 cases) or the immunoreactivity was distributed homogeneously within the lymphoid population. Endothelial cells were also intensively positive for tyrosine p-STAT-3 and served as an internal positive control in the negative cases.

SOCS-3 expression was cytoplasmic and was detected in 34/35 (97.1%) of the examined cases (Table 4, Figure 3(f)). In a significant proportion of the cases (14/34, 41.2%) SOCS-3 immunoreactivity was detected in the PCs (Figure 3(f)), whereas in the remaining cases the staining was homogeneously distributed throughout the lymphoid tissue.

VEGF expression was cytoplasmic and was observed in all examined cases (41/41, 100%) (Table 4). Staining was more pronounced in the PCs in 18 cases (18/41, 43.9%, median H-score within the PCs 90 versus 25 outside the PCs, matched pairs signed-rank test, *P* < 0.0001, Figures 2(h), 3(g), and 3(h)).

### 3.3. Associations among IL-6, IL-8, CXCR2, Tyrosine p-STAT-3, SOCS-3, VEGF Expression as well as with Microvascular Characteristics and Clinicopathological Parameters

VEGF H-score was negatively correlated with MVD (*R* = − 0.3291, *P* = 0.0500) and shape factor (*R* = − 0.4726, *P* = 0.0036). When, however, VEGF H-score and microvascular characteristics were analyzed within the PCs, no significant relationship was documented (*P* > 0.10). The remaining correlations among IL-6, IL-8, CXCR2, SOCS-3, p-STAT-3 immunoreactivity and microvessels' characteristics were not significant (*P* > 0.10).

**Figure 4:**
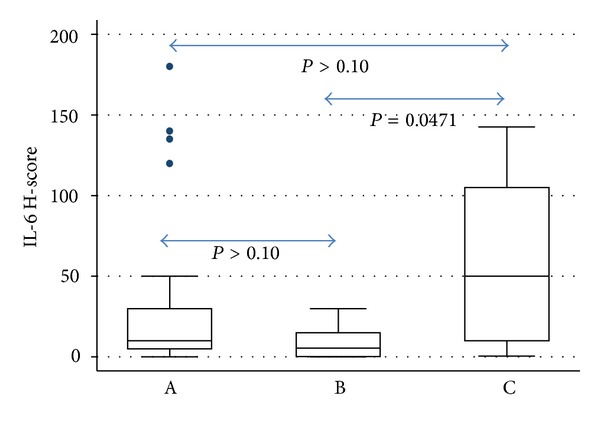
Box plot illustrating the correlation between IL-6 H-score and Binet stage. IL-6 was higher in stage C, followed by stage A, whereas stage B cases displayed the lower levels of IL-6 H-score. Post hoc analysis indicated that the only significant difference was that between stages B and C (*P* = 0.0471, Tukey HSD method).

IL-6 H-score was associated with Binet stage (Kruskal-Wallis ANOVA, *P* = 0.0433, Figure 4) being higher in stage C, followed by stage A, whereas stage B cases displayed the lower levels of IL-6 H-score. Post hoc analysis indicated that the only significant difference was that between stages B and C (*P* = 0.0471, Tukey HSD method). Moreover, it was inversely correlated with white blood cell count (WBC) (*R* = − 0.03084, *P* = 0.0311) and absolute lymphocyte count (ALC) (*R* = − 0.3144, *P* = 0.0278).

SOCS-3 H-score was adversely associated with the presence of splenomegaly and lymphadenopathy (Mann-Whitney* U* test, *P* = 0.0275 and *P* = 0.0504 resp.).

VEGF H-score was negatively correlated with Binet stage (Mann-Whitney* U* test, A versus B/C, *P* = 0.0385). Inverse relationships were also observed between Binet stage (A versus B/C) and minor axis length (*P* = 0.0433), area (*P* = 0.0303), major axis length (*P* = 0.0549), as well as perimeter (*P* = 0.0656) the latter two being of borderline significance.

The remaining associations between IL-6, IL-8, CXCR2, SOCS-3, p-STAT-3, and VEGF immunoreactivity as well as microvascular characteristics with patients' clinical and laboratory findings were not significant.

### 3.4. Associations between IL-6, IL-8, CXCR2, Tyrosine p-STAT-3, SOCS-3, VEGF Expression, Microvascular Characteristics, and Ki-67 Index as well as Fas, Fasl, and c-Flip Expression

A significant positive correlation emerged between tyrosine p-STAT-3 and proliferation rate (i.e., Ki-67 index) (Spearman's correlation coefficient, *R* = 0.3713, *P* = 0.0217). The respective relationship between IL-6 H-score and Ki-67 index was negative (Spearman's correlation coefficient, *R* = −0.3514, *P* = 0.0356). SOCS-3 H-score was inversely correlated with FasL expression within the PCs (Spearman's correlation coefficient, *R* = −0.4544, *P* = 0.0385).

The remaining correlations between the investigated molecules and Ki-67 index, Fas, FasL, and c-FLIP were not significant.

### 3.5. Survival Analysis

**Figure 5:**
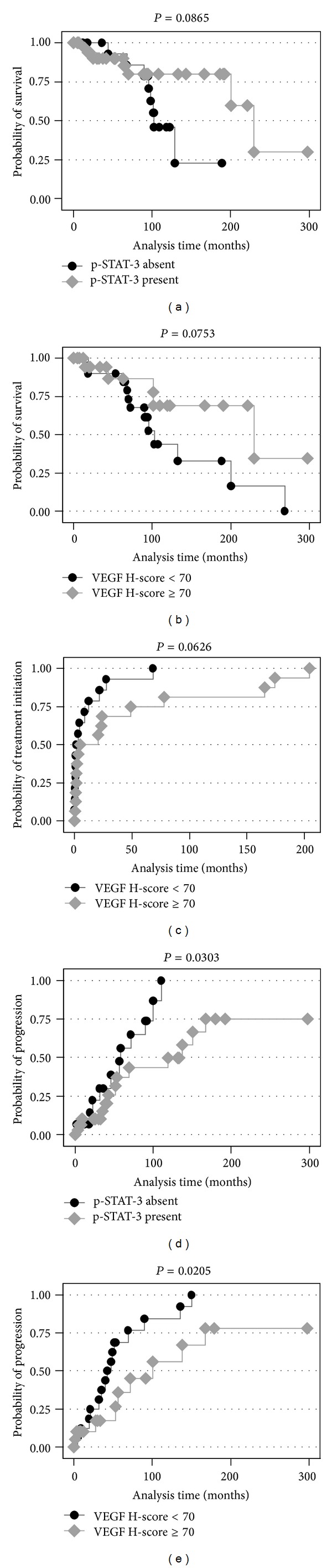
(a, b) Kaplan Meier survival curves for OS according to tyrosine p-STAT-3 immunoreactivity (a) and VEGF H-score (b). (c) Kaplan Meier failure curves for TFT according to VEGF H-score. (d, e) Kaplan Meier failure curves for TTP according to tyrosine p-STAT-3 immunoreactivity (d) and VEGF H-score (e).

In univariate analysis, Binet stage (*P* < 0.0001), the presence of* ATM *(*P* < 0.0001) and* p53 *(*P* = 0.0001) mutations and the presence of bulky lymphadenopathy (*P* = 0.0113) and B symptoms (*P* = 0.0045) were adversely correlated with OS. Moreover, the presence of tyrosine p-STAT-3 immunoreactivity (*P* = 0.0865, Figure 5(a)) and increased VEGF H-score (*P* = 0.0753, Figure 5(b)) was marginally correlated with increased OS.

Regarding TFT, Binet stage (*P* = 0.0025), the presence of* p53 *(*P* = 0.0420) mutations and the presence of increased WBC (*P* = 0.0129) and bulky lymphadenopathy (*P* = 0.0051) emerged as adverse prognosticators. Increased VEGF H-score also marginally correlated with lower TFT (*P* = 0.0626, Figure 5(c)).

With regard to TTP, advanced Binet stage (*P* = 0.0010), the presence of* ATM *(*P* = 0.0070) and* p53 *(*P* = 0.0188) mutations along with the presence of bulky lymphadenopathy (*P* = 0.0252) and B symptoms (*P* = 0.0682) exerted an adverse influence, the latter being of marginal significance. Importantly, tyrosine p-STAT-3 positivity (*P* = 0.0303, Figure 5(d)) and increased VEGF H-score (*P* = 0.0205, Figure 5(e)) were correlated with longer TTP. The median TTP for those patients who displayed p-STAT-3 immunoreactivity was 137.766 months, whereas the respective value for those patients who were negative for tyrosine p-STAT3 was 58.26 months. Accordingly, the median TTP for those patients who displayed increased VEGF H-score (≥70) was 100.3 months, whereas the respective value for those patients who displayed decreased VEGF H-score (<70) was 42.36 months.

In multivariate analysis, the only parameter that correlated with OS or TFT was Binet stage (Table 5, models A and B). Finally, advanced Binet stage and the absence of p-STAT-3 expression were correlated with shorter TTP (Table 5, model C). VEGF effect was marginal in this regard.

## 4. Discussion

A number of studies pursued during the last few years have looked into the potential pathophysiological role of angiogenesis in CLL, but the results have often been inconsistent (rev. in [2]). The present investigation differs from the previous ones in several aspects. First, angiogenesis is examined in lymph nodes rather than in the bone marrow. Second, we have assessed the microvascular network by estimating not only MVD and TVA but also various parameters reflecting microvessel shape and caliber. Third, we evaluated the intracellular levels of proangiogenic cytokines IL-6 and IL-8 which are known to be elevated in the serum of CLL patients [28]. Fourth, we have explored for the first time the* in situ* expression of CXCR2—the prime functional chemokine receptor mediating endothelial cell chemotaxis [29], as well as of ^tyr^p-STAT-3/SOCS-3 axis modulating cytokine signaling [30].

The vast majority of our cases (81.6%) were devoid of IL-8 and CXCR2 immunoreactivity rendering IL-8/CXCR2 axis unlikely to conduct CLL pathogenesis or angiogenesis. An earlier study, however, has documented constitutive IL-8 mRNA in purified B-CLL cells obtained from 17 patients [31]. On the contrary, IL-6 was identified in almost all cases, in harmony with Biondi et al. [32] who reported constitutive expression of IL-6 gene transcripts in CLL. IL-6 levels in our cohort were significantly higher in Binet stage C than in stage B, but they were unrelated to VEGF expression, as reported by Molica et al. [33] and correlated inversely with WBC or ALC. Increased IL-6 serum levels have also been documented in high-stage CLL by Fayad et al. [34]. It is unclear, however, whether the source of elevated serum IL-6 is solely the neoplastic cell in CLL, since normal peripheral blood mononuclear cells are also known to produce IL-6 (rev. in [34, 35]). In addition, it has long been shown that this molecule is also able to inhibit the growth of CLL cells [36], thus providing an explanation for the negative correlation between IL-6 and Ki-67 obtained in the present investigation. Serum IL-6 levels have been reported to confer an unfavorable prognosis in CLL (rev. [37]), without constituting an independent prognostic factor [38], an effect which could not be substantiated in our study. Of note, serum cytokine levels may not necessarily be indicative of the cytokine profiles within bone marrow or lymph nodes where CLL cells receive the influence of important growth and survival signals. Therefore, expression studies are essential in order to dissect whether their elevated levels are related to CLL biology or merely represent a consequence of other stimuli within CLL microenvironment [28].

SOCS-3 protein was identified in the majority of our cases (97.1%) and was unrelated to IL-6 expression, confirming the results of a recent study investigating the serum SOCS-3 mRNA levels in a small number of CLL patients [38]. The absence of the expected negative correlation between IL-6 and SOCS-3 may be attributed to the induction of SOCS-3 by a variety of cytokines, for example, IL-10 and lipopolysaccharide, raising the issue of IL-6 regulation by pathways other than JAK/STAT. In our study, SOCS-3 expression tended to be associated with the absence of adverse prognosticators, such as bulky lymphadenopathy or splenomegaly, creating the impression of behaving rather like a tumor suppressor [39]. This is strengthened by the inverse correlation between SOCS-3 and FasL expression within PCs, the latter being a mechanism adopted by CLL cells to avoid immunosurveillance [26]. In support of this role, SOCS-3 overexpression reportedly diminishes proliferation of classical Hodgkin lymphoma cell lines [40], whereas its loss has been postulated to predispose for the emergence of a more aggressive disease [39, 41].

Most published information regarding STAT-3 activation in CLL focuses on its serine 727 phosphorylation, which constitutes a hallmark of CLL regardless of cytogenetic abnormalities and clinical characteristics (rev. in [10, 12]). In the present investigation, we sought to determine for the first time the significance of STAT-3 phosphorylation on tyrosine 705, which is described to be transiently induced by IL-6 in CLL cells [10]. We identified ^tyr^p-STAT-3 in two-thirds of CLL cases, albeit at low levels. p-STAT-3 showed a predilection for the PCs and correlated positively with Ki-67. On the basis of these findings, it is reasonable to assume that, although perhaps biologically less relevant than serine p-STAT-3, tyrosine p-STAT-3 may exert a proliferative effect on CLL cells. Application of tyrosine kinase inhibitors of JAK/STAT pathway in Hodgkin lymphoma cell lines was accompanied by a strong antiproliferative effect and enhanced death [40], lending support to our hypothesis. Intriguingly, the presence of tyrosine p-STAT-3 was predictive of longer TTP or marginally OS, the former effect which remained in multivariate analysis. Such conflicting data reflects the complexity of STAT-3, functioning both as a tumor suppressor or an oncogene, also documented in nonhematopoietic tumors [42–44]. Relevant to this notion is the fact that STAT-3 upregulates and downregulates the expression of miRs in CLL cells [42], acting as a mediator of both suppression and activation of transcription.

Remarkably, p-STAT-3 and SOCS-3 were simultaneously present in the PCs in 77.3% of cases. Given that SOCS-3 is a negative regulator of JAK/STAT pathway [30], one would expect the two proteins to be mutually exclusive. This finding is not without precedence, having been previously noted in nonhematopoietic tumors [42, 44, 45] and several hematopoietic cell lines [40, 46]. In this context, it has been hypothesized that the activation of STAT-3 may be due to kinases that cannot be suppressed by SOCS-3 or that tumor cells may develop strategies to bypass the negative regulation mediated by SOCS-3 [40].

Interestingly, both the extent of microvasculature and microvessel caliber was significantly lower within PCs as compared to non-PC areas. In fact, microvessels were more numerous at the periphery of the PCs, as reported by Frater et al. [47] in the bone marrow. On the contrary, VEGF expression was enhanced within the PCs which accounts for the apparently paradoxical inverse correlation between the density of microvessels and VEGF we observed. Indeed, when both VEGF and microvascular parameters were analyzed within PCs no significant relationships emerged. Therefore, although VEGF is undoubtedly a crucial regulator of angiogenesis and its inappropriate early induction is considered to underlie increased angiogenesis in CLL [48], the latter is dependent upon the concerted action of many angiogenic molecules besides VEGF, such as Ang2. In the bone marrow infiltrated by CLL cells, Ang2 and not VEGF correlated with MVD [48]. The distinct topography of VEGF and vascular hot spots in CLL may portray a normal sequence of events since the low vascularity combined with the retarded blood flow, illustrated by the preponderance of rounder vessel sections within PCs, creates a hypoxic microenvironment triggering the increased VEGF expression [47]. This scenario fits well with the enhancement of VEGF and Ang2 protein secretion by CLL cells subjected to hypoxia [48] and with the expression of HIF-1a, the main transcriptional regulator of VEGF by CLL cells [47]. Following this line of argument, the hypoxic microenvironment within PCs may be the common denominator of p-STAT-3 and SOCS-3 upregulation given that they are both functionally linked to HIF-1a [49, 50].

We were unable to elicit any association between microvascular characteristics and laboratory parameters, survival or treatment response, as in the study of Szmigielska-Kapłon et al. [51] or Frater et al. [47] who, however, have focused only on MVD. The correlation between MVD and clinical stage in CLL has been a subject of controversy with some studies documenting a positive relationship [51, 52], while others failing to observe such a relationship [53, 54]. These observations along with the fact that MVD has been reportedly higher in unmutated CLL [48] point towards deregulation of angiogenesis being a rather precocious event facilitating the evolution of CLL. Noteworthy, microvessel caliber was higher in early stage CLL in our series.

An intriguing finding of the present investigation is the inverse relationship of intracellular VEGF levels with Binet stage. VEGFR1 levels have also been reported to decrease with the duration of the disease [55], implying that VEGF upregulation may be an early event during lymphomatogenesis. On the contrary, serum VEGF has been shown to increase in parallel with disease stage and the risk of progression [56]. Therefore, not surprisingly, we documented a favorable prognostic effect of VEGF expression levels with regard to *ΤΤ*P which remained in multivariate analysis, albeit of marginal significance. Aguayo et al. [57] also reported lower VEGF plasma levels to be predictive of shorter survival times in early-stage CLL as well as in *β*2-microglobulin negative cases. In addition, peripheral VEGF levels inversely correlated with WBC and ALC. Whether this unexpected reverse correlation of serum or tissue VEGF with clinical outcome is linked to the expression of 43KD isoform, as implied by Aguayo et al. [57], remains to be proven.

The fact that established prognosticators in CLL patients (i.e., Binet stage, elevated LDH, 17pdel) turned out to be significant in our survival analysis advocates that out cohort was representative. However, the small number of events precluded the introduction of more parameters in multivariate analysis and relates to the absence of prognostic significance assigned to ZAP-70 expression. A further drawback might be the fact that IgVH mutational status and several prognostically important chromosomal abnormalities could not be assessed in the entire cohort.

In summary, the present investigation constitutes the first attempt to elucidate the significance of angiogenesis and proangiogenic cytokines, IL-6, IL-8, and VEGF as well as p-STAT-3 and SOCS-3 in lymph nodes involved by CLL. Overall, our results fail to establish that microvascular parameters and Th2-cytokines are related to each other or to prognosis. A novel observation is that PCs appear to be rather hypoxic due to the lower extent of microvascular network and to hemorrheologic conditions, thus presumably driving the expression of VEGF, tyrosine p-STAT-3, and (to a lesser extent) SOCS-3 in these regions. Finally, we bring forward the favorable prognostic effect of VEGF expression at the tissue level, as reported in the peripheral blood, as well as of p-STAT-3 positivity consistent with its tumor suppressive effect. These observations warrant verification in large prospective series to validate the role of VEGF and p-STAT-3 as outcome predictors in CLL patients.

## Figures and Tables

**Table 1:** Patients' characteristics at diagnosis.

Characteristic	
Age, median (range)	58 (36–78)

	*n* (%)

Male	40 (64.5)
Binet Stage	
A	37 (59.7)
B	17 (27.4)
C	8 (12.9)
B-symptoms	6 (11.10
Bulky lymphadenopathy	7 (13.2)
Splenomegaly	17 (31.5)
*Η*b level (<11 gr/dL)	8 (14)
Platelet count (<100 × 10^9^/L)	3 (5)
Hyperglobulinemia	4 (7.4)
Hypoglobulinemia	12 (22.6
*Β*2 microglobulin (abnormal)	11 (73.3)
LDH > UNL*	18 (32.7)
Absolute lymphocyte count (>10 × 10^9^/L)	29 (51)
CD38 (+)	27 (55.1)
ZAP-70 (+)	27 (51.9)
IGVH unmutated	13 (81.3)
*11 q del *	1 (5.6)
*17 p del *	2 (9.1)

*UNL: upper normal limit.

**Table 2:** Characteristics of primary antibodies used in immunohistochemical analysis.

Protein	Number of cases	Clone	Company	Catalog number	Raised in	Positive controls	Antigen retrieval method	Dilution and incubation time for immunohistochemistry
IL-8	51^†^	polyclonal	Invitrogen Corporation, Camarillo, CA	AHC 0881	rabbit	Normal tonsillar tissue	pH 6 (low)	1 : 50, 18 h 4°C

IL-6	51^†^	polyclonal	Santa Cruz Biotechnology, Santa Cruz, CA	SC 1265	goat	Normal tonsillar tissue	pH 6 (low)	1 : 50, 18 h 4°C

SOCS-3	35^†^	polyclonal	Santa Cruz Biotechnology, Santa Cruz, CA	SC 9023	rabbit	Cholangiocarcinoma	pH 6 (low)	1 : 100, 18 h 4°C

CXCR2	51^†^	monoclonal	R and D Systems, Abingdon, England	MAB 331	mouse	Normal tonsillar tissue	pH 9 (high)	1 : 100, 18 h 4°C

VEGF [recognizes 165, 189, 206 aa isoforms of VEGF]	41*	monoclonal	Pharmingen BD Company, San Diego, CA	clone G153-694	mouse	Glioblastoma	pH 6 (low)	1 : 40, 18 h 4°C

p-STAT3 [specific at site Tyrosine 705]	51^†^	monoclonal	Cell Signaling Technology Inc., Boston, MA, USA	D3A7 XP	rabbit	Human breast cancer	pH 6 (low)	1 : 100, 18 h 4°C

CD34	54^†^	monoclonal	Dako, Carpinteria, CA	clone QBEnd/10	mouse	Kaposi sarcoma	pH 9 (high)	1 : 50, 30 min room temperature

*21 cases were excluded due to loss of antigen preservation.

^†^In the remaining cases, staining information was not available due to exhaustion of the available tissue.

**Table 3:** Microvascular characteristics.

	Within PCs	Outside PCs	Overall
MVD	12 (3–45)	25 (10–62)	58 (15–247)
TVA (*μ*m^2^)	341 (36–1913)	768 (328–3646)	1778 (494–11942)
Major axis length (*μ*m)	6.7 (3–15.2)	9.1 (4.8–14.2)	7.33 (4.92–13.76)
Minor axis length (*μ*m)	4.3 (2.1–12.4)	6.02 (2.8–10.9)	5.13 (2.99–10.88)
Perimeter (*μ*m)	13.6 (6.2–32.7)	18.1 (9.5–3.6)	15.28 (9.1–28.1)
Shape factor	0.81 (0.78–0.88)	0.8 (0.78–0.83)	0.81 (0.74–0.87)
Compactness	20.6 (19.2–25.2)	21.1 (17.3–22.7)	20.65 (17.6–29.1)
Feret diameter (*μ*m)	11.7 (9.5–14.5)	11.8 (9.5–14.3)	10.3 (8.17–21.52)
Area (*μ*m^2^)	26.3 (12.02–63.1)	35.5 (15.9–58.8)	29.64 (16.07–55.61)

**Table 4:** IL6, IL8, CXCR2. and VEGF immunostaining results.

Immunohistochemical staining (H-score)	Percentage of positive cases	Median value in positive cases	Range	
IL6	96.08%	10	0	180
IL8	9.80%	4.5	0	75
CXCR2	13.21%	30	0	60
SOCS-3	97.1%	67.5	0	175
Tyrosine p-STAT-3	66.67%	1	0	16
VEGF	100%	70	0.75	212

**Table 5:** Multivariate Cox's proportional hazards estimations according to OS (model A), TFT (model B), and TTP (model C).

	Parameter	Hazard Ratio (HR)	*P* value	95% CI of HR	
A	Binet stage (A versus B versus C)	2.871	0.048	1.011	8.151
	B symptoms (absence versus presence)	4.203	0.088	0.806	21.913

B	Binet stage (A versus B versus C)	2.199	0.001	1.369	3.534

C	Binet stage (A versus B versus C)	4.271	0.003	1.651	11.049
	B symptoms (absence versus presence)	4.188	0.061	0.935	18.764
	Tyrosine p-STAT-3 expression (absence versus presence)	0.202	0.024	0.050	0.813
	VEGF H-score (<70 versus ≥70)	0.375	0.078	0.117	1.198
